# Alleviation of Cadmium and Nickel Toxicity and Phyto-Stimulation of Tomato Plant L. by Endophytic *Micrococcus luteus* and *Enterobacter cloacae*

**DOI:** 10.3390/plants11152018

**Published:** 2022-08-03

**Authors:** Ibrahim H. Badawy, Ahmed A. Hmed, Mahmoud R. Sofy, Alshymaa Z. Al-Mokadem

**Affiliations:** 1 Botany and Microbiology Department, Faculty of Science, Al-Azhar University, Cairo 11884, Egypt; badawyibrahim0@gmail.com (I.H.B.); ahmed_hmed@azhar.edu.eg (A.A.H.); 2 Botany Department, Women’s College, Ain Shams University, Cairo 11566, Egypt; alshymaa.almokadem@women.asu.edu.eg or

**Keywords:** heavy metals, bioconcentration factor, translocation factor, antioxidant defenses, specific regulatory defense genes

## Abstract

Cadmium (Cd) and nickel (Ni) are two of the most toxic metals, wreaking havoc on human health and agricultural output. Furthermore, high levels of Cd and Ni in the soil environment, particularly in the root zone, may slow plant development, resulting in lower plant biomass. On the other hand, endophytic bacteria offer great promise for reducing Cd and Ni. Moreover, they boost plants’ resistance to heavy metal stress. Different bacterium strains were isolated from tomato roots. These isolates were identified as *Micrococcus luteus* and *Enterobacter cloacae* using 16SrDNA and were utilized to investigate their involvement in mitigating the detrimental effects of heavy metal stress. The two bacterial strains can solubilize phosphorus and create phytohormones as well as siderophores. Therefore, the objective of this study was to see how endophytic bacteria (*Micrococcus luteus* and *Enterobacter* *cloacae*) affected the mitigation of stress from Cd and Ni in tomato plants grown in 50 μM Cd or Ni-contaminated soil. According to the findings, Cd and Ni considerably lowered growth, biomass, chlorophyll (Chl) content, and photosynthetic properties. Furthermore, the content of proline, phenol, malondialdehyde (MDA), H_2_O_2_, OH, O_2_, the antioxidant defense system, and heavy metal (HM) contents were significantly raised under HM-stress conditions. However, endophytic bacteria greatly improved the resistance of tomato plants to HM stress by boosting enzymatic antioxidant defenses (i.e., catalase, peroxidase, superoxide dismutase, glutathione reductase, ascorbate peroxidase, lipoxygenase activity, and nitrate reductase), antioxidant, non-enzymatic defenses, and osmolyte substances such as proline, mineral content, and specific regulatory defense genes. Moreover, the plants treated had a higher value for bioconcentration factor (BCF) and translocation factor (TF) due to more extensive loss of Cd and Ni content from the soil. To summarize, the promotion of endophytic bacterium-induced HM resistance in tomato plants is essentially dependent on the influence of endophytic bacteria on antioxidant capacity and osmoregulation.

## 1. Introduction

The industrial revolution expanded human activity, which has facilitated the transport of several possible pollutants, such as cadmium and/or nickel, from the earth’s crust to various environmental compartments [1].

Heavy metals (HMs) are high-density transition metals that can be toxic at low concentrations [2]. HMs accumulation in plants and soil has increased the risk to human health in recent decades; thus, HMs are potentially hazardous to the environment [3]. Furthermore, HMs stifle plant growth by inhibiting various functions, including photosynthesis, respiration, glucose metabolism, and water relations [4].

Heavy metal ion contamination by cadmium (Cd) and nickel (Ni) contaminants is a worldwide issue caused by human, technological, and geological activities [5]. Cd concentrations naturally range from 0.01 to 0.7 mg kg^−1^ in agricultural soils [6]. On the contrary, Ni concentrations, according to the World Health Organization (WHO), range from 15 to 30 mg kg^−1^ in soil [5].

On the other hand, the higher concentration of heavy metals, the greater the phytotoxicity, which inhibits nutrient absorption, disrupts the photosystem, compromises membrane integrity, alters nitrogen metabolism, and accelerates necrosis and chlorosis, and senescence [7]. Additionally, Cd and Ni stress deform the chloroplast structures [8], resulting in decreased chlorophyll biosynthesis [9]. Cd and Ni toxicity significantly hinders the absorption, uptake, and translocation of Cu, Zn Fe, and Mn in different plant species, resulting in plant nutrient shortage [10]. Further, the increased concentration of Cd and Ni causes oxidative stress by over-forming reactive oxygen species (ROS) [5], decreases the electron supply in the photosynthetic electron transport chain, and damages DNA molecules, lipids, and proteins [11]. Metal-induced oxidative damage requires plants to develop several effective management strategies, and among them, the antioxidant defense mechanism plays an important role [12].

A plant growth-promoting rhizobacteria (PGPR) establishes beneficial ecological relationships with plants to stimulate their growth through direct and indirect interactions with them. The former involves biological nitrogen fixation, organic acid and siderophores production, regulation of phytohormone synthesis, and aminocyclopropane-1- carboxylic acid deaminase enzyme activity [13]. As indirect mechanisms, phytopathogens are inhibited through competition for nutrients, antibiosis by secondary metabolites, lytic enzymes, induction of plant immune responses, and improved soil physicochemical characteristics [14]. The presence of plant growth-promoting rhizobacteria (PGPR) may enable plants to withstand heavy metal stress under challenging conditions and apply other adaptations. There is a large diversity of microbes in the rhizosphere. Even so, some plant growth-promoting bacteria (PGPBs) encourage plant growth and improve their tolerance to heavy metals [9]. Typically, these relationships are symbiotic, which means bacteria live inside plants without damaging them [15]. Rhizobacteria that promote plant growth in the presence of heavy metals and physiological and biochemical mechanisms can be used to improve nutrient availability and absorption in plants [16].

Tomato (*Solanum lycopersicum* L.) is commonly grown in the subtropics, tropics, and warm temperate zones. It has long been recognized as an important vegetable crop enjoyed by people worldwide [17].

However, tomatoes are more vulnerable to environmental pressures, including floods, salt, drought, and heavy metals such as Cd and Ni. According to the author’s knowledge, only a few studies have looked at Cd and Ni-induced morpho-biochemical and physiological alterations in tomato plants and their amelioration utilizing various growth modulators. As a result, the present research was conducted to see if endophytic bacteria may help tomato plants tolerate severe Cd and Ni poisoning. It was hypothesized that using *Micrococcus luteus* and *Enterobacter cloacae* may reduce Cd and Ni absorption in tomato plants and would ameliorate Cd and Ni-induced oxidative damage. The study’s main goals were to:

(i) Evaluate the efficiency of *Micrococcus luteus* and *Enterobacter cloacae* when applied with Cd and Ni on tomato plants.
(ii) Assess the role of *Micrococcus luteus* and *Enterobacter cloacae* in accelerating morphological, antioxidant enzyme activities, biochemical, and physiological in tomato plant against Cd and Ni stress.

Furthermore, these results will add to our knowledge of the processes behind the improved tolerance to Cd and Ni in tomatoes when grown with *Micrococcus luteus* and *Enterobacter cloacae* growth modulators.

## 2. Material and Methods

### 2.1. Isolation of Endophytic Bacteria from Roots of Tomato Plant 

#### 2.1.1. Collection of Plant Samples

Seventeen samples of roots from tomato (*Solanum lycopersicum*) plants were randomly collected from tomato fields in the governorate of Kafr El-Sheikh (30°56′45″ N, 42°06′31″ E) in Egypt. In sterile polyethylene bags, all collected samples were placed and transported in ice boxes to the plant viruses and bacteriophage laboratory at the Faculty of Science of the Botany and Microbiology Department, Al-Azhar University, Cairo, and stored at 4 °C.

#### 2.1.2. Surface Sterilization of Samples

The sample roots were cleaned adequately with sterile distilled water (DW) to eliminate any remaining dirt. The samples were then surface sterilized for 3 min with 75% ethanol, then by a 10-min wash in 2.5% sodium hypochlorite and a five-times wash in sterile DW. To determine the effectiveness of surface sterilization, 100 mL of the final rinse was plated on nutrient agar plates (NA, DifcoTM, BD, Franklin Lakes, NJ, USA) and incubated at 30 °C for 48 h. Subsequently, the samples were dried aseptically using sterile paper [18].

#### 2.1.3. Isolation of Endophytic Bacteria

The approach provided by Etesami et al. [19] was used to isolate endophytic root bacteria. With a sterile mortar and pestle, fragments of root materials were mashed in 5 mL of 12.5 mM potassium phosphate buffer (pH 7.0). Serial dilutions of root tissue extract were then performed in potassium phosphate buffer (pH 7.0). Following the series preparation from the samples, 0.1 mL of each dilution of 10^−4^ to 10^−7^ was distributed on NA plates. After autoclaving, the media were treated with 10 mg L^−1^ of fungicidin to inhibit the development of endophytic fungi. The number of colonies emerging on the plates was counted after all plates were inverted and put in an incubator for three days at 30 °C. The weight of fresh root CFU g^−1^ was used to calculate the amount of isolated endophytic bacteria. Subculture was used to carry out the purification processes of these isolates in the same medium. Finally, the bacterial isolates were pooled and kept in a refrigerator at 4 °C for further analysis according to phenotypic traits (colony morphology, color, growth rate, shape, and motility) and Gram-staining. Furthermore, the Isolates were kept at −80 °C in nutritional broth (NB, Difco^TM^, BD, Franklin Lakes, NJ, USA) containing 20% glycerol to ensure long-term stability.

#### 2.1.4. Morphological and Biochemical Characteristics of the Isolates

The traditional gram staining approach evaluated cell morphology [20]. However, biochemical tests were performed for urease activity, catalase, oxidase, citrate utilization, nitrate reduction, hydrogen sulfide generation, indole formation, methyl red, and Voges-Proskauer assays.

#### 2.1.5. Molecular Characterization of Bacterial Isolates

The used kit’s instruction manual extracted genomic DNA from the selected bacterial isolates (QIA amp mini kit cat number, 51304). According to James [21], the 16S rDNA region was amplified (approximately 1500 bp) by polymerase chain reaction (PCR) with the forward primer 27F (5′-AGAGTTTGATCCTGGCTCAG-3′) and the reverse primer 1495R (5′-CTACGGCTACCTTGTTACGA-3′). The PCR components were prepared in 50 (μL) volumes containing 0.5 μM of primer, 5 μL of the 10X PCR buffer (500 mM KCl; 100 mM Tris–HCl, 15 mM MgCl_2_, pH 8.3), 200 mM deoxyribonucleotide triphosphate, and 1 (μL) of the extracted DNA and 1 U Taq DNA polymerase. The initial denaturation at 94 °C for 5 min was followed by 35 cycles of denaturation at 94 °C for 1 min annealing at 50 °C for 1 min, and elongation at 72 °C for 1 min, followed by a final extension at 72 °C for 5 min in the TProfessional Basic Thermocycler PCR system. After the identification of the amplicon on 1% agarose gel using a DNA size marker (UMR-100) and subsequent purification with a PCR purification kit (Tianwei), the amplicon was identified by horizontal electrophoresis.

#### 2.1.6. Sequence Alignments and Phylogenetic Analyses

DNAMAN software (Madison, WI, USA) and the clustalw (Ver. 1.74) program were used to align sequences multiple times [22]. Nucleotide distances were calculated using Molecular Evolutionary Genetics Analysis (MEGA) software (Ver. 11.0, State College, PA, USA) and the Jukes and Cantor method [23] for the correction of superimposed substitutions [24]. Phylogenetic relationships were assessed using the Unweighted Pair Group Method with Arithmetic Mean (UPGMA) in DNAMAN software and Neighbour Joining (NJ) in MEGA 4.0 software, as well as a bootstrap analysis (1000 replicates) to assess the reliability of the phylogenetic tree.

#### 2.1.7. Screening of the Most Potent Growth-Promoting Endophytic Bacterial Isolates

A total of 63 endophytic bacterial isolates were investigated in vitro for their potential to promote plant development, including the generation of indole 3-acetic acid (IAA) by HPLC [25], nitrogen fixation [26], phosphate solubilization activity [27], biofilm formation activity [28], 1-aminocyclopropane-1-carboxylic acid (ACC) deaminase activity [29], and root colonization ability [30].

#### 2.1.8. Determination of the Tolerance of Endophytes Strains to Heavy Metal Stress

Fourteen endophytic bacterial isolates demonstrating the most significant plant growth stimulation (from the previous experiment) were tested for Cd and Ni stress tolerance. The growth of the strains was tested on NA plates with various cadmium chloride (CdCl_2_) and nickel chloride (NiCl_2_. 6H_2_O), concentrations (0.25, 0.5, 1.0, 1.5, and 2.0 µM for each metal). Growth was estimated after two days of incubation at 30 °C in the dark [31]. 

#### 2.1.9. Bacterial Suspension Inoculation and Treatments

The fresh inoculum of each one of the endophytic bacterial isolates was prepared as follows. Initially, endophytic bacterial isolates were recultured in NA medium. Then, the 100 mL flask containing 20 mL of sterilized NB culture medium was inoculated with bacterial isolates. Cultures were incubated at 30 °C for 48–72 h on a shaker with a rotation speed of 120 rpm until the mid-logarithmic phase was attained 5 × 10^8^ CFU mL^−1^. The following procedures were followed to make fresh inoculum from each endophytic bacterial isolate: Endophytic bacteria isolates were first recultured in an NA medium. The bacterial isolates were then injected into a 100 mL flask containing 20 mL of sterilized NB culture medium. Cultures were cultured at 30 °C for 48–72 h on a shaker with a rotation speed of 120 rpm until the mid-logarithmic phase was reached, measured at 5 × 10^8^ CFU mL^−1^ using a 0.5 McFarland turbidity standard [32]. After growing in NB culture medium, the bacteria were collected by centrifugation (7000× *g* for 10 min), washed with phosphate buffer, and suspended in phosphate buffer. The tomato seeds were soaked in the bacterial solution, according to Rajendra et al. [33]. 

### 2.2. Plant Material

The seeds of tomato (*Solanum lycopersicum*) were purchased from ARC, Giza, Egypt. First, the healthy seeds of similar size were surface-sterilized for ten minutes with a 1% sodium hypochlorite solution before rinsing with DW. Next, the seedlings were moved into well-maintained 40-cm pots 7 days after seeding. It comprised a sterile soil mixture of 30% peat moss, 34% clay, and 36% sand, with the temperatures in the day/ night at 27/21 °C, relative humidity at 53–57%, light intensity at 100–200 μmol m^−2^ s^−1^, and ambient CO_2_ levels during the 300–410 μmol mol^−1^. The characteristics of the soil were as follows: pH 7.2; electrical conductivity (EC) 1.29 dS m^–1^; anions (meq L^–1^), SO_4_ 5.67, Cl^–^ 4.59, HCO_3_^−^ 1.61, and cations (meq L^−1^), Mg^2+^ 1.72, Ca^2+^ 4.30, K^+^ 0.36, Na^+^ 4.69. Seedling roots were applied with 50 μM Cd and Ni at the same time. The Cd and Ni concentrations were chosen based on the previous study [34,35]. Cd and Ni were treated in the form of cadmium chloride (CdCl_2_) and nickel chloride (NiCl_2_. 6H_2_O), respectively. Plants were irrigated twice with heavy metal (Cd and Ni) and once with water to prevent metal accumulation. After 14 days of growth, the plants were split into nine groups. Each group is made up of six replicate pots. Each treatment had five replications in a complete block randomized design (CRD). The groups were divided into:

T1: (Control with tap water)

T2: *Micrococcus luteus*. 

T3: *Enterobacter cloacae*.

T4: 50 μM Cadmium chloride (CdCl_2_) (Cd)

T5: *Micrococcus luteus* + 50 µM Cd

T6*: Enterobacter cloacae* + 50 µM Cd

T7: 50 µM nickel chloride (NiCl_2_. 6H_2_O) (Ni)

T8: *Micrococcus luteus* + 50 µM Ni

T9*: Enterobacter cloacae* + 50 µM Ni

### 2.3. Measurement of Growth Parameter

Five samples were collected from various treatments for morphological characteristics (shoot length and root length, fresh and dry weight of shoot and root). A meter scale determined the shoot and root lengths. In addition, six shoot and root samples were collected randomly from each treatment to analyze physiological parameters in roots and leaves and specific regulatory defense genes in leaves. The fresh sample dried to a steady dry weight at 65 °C.

### 2.4. Chlorophyll Content and Photosynthetic Characteristics

To determine chlorophyll SPAD content in tomato leaves, a SPAD chlorophyll meter was used [17]. The portable photosynthetic technique (LICOR 6400, LICOR, Lincoln, NE, USA) was used to determine the photosynthetic properties (stomatal conductance (gs), net photosynthetic rate (PN), transpiration rate (E), and intercellular CO_2_ concentration (Ci)) in the extended upper part plant leaves in each treatment. Relative humidity, photosynthetic photon flux density, air temperature, and CO_2_ concentration were maintained at 85%, 800 μmol mol^−2^ s^−1^, 25 °C, and 600 ppm [17].

### 2.5. Determination of Stress-Induced Biomarkers

#### 2.5.1. Total Proline and Phenol Content 

The free proline concentration in leaf tissue was measured using acid ninhydrin produced with glacial acetic acid and phosphoric acid, as Bates et al. [36] described. The leaf’s free phenols content was measured using sodium carbonate solution, and the Folin–Ciocalteu reagent was then read at 765 nm [37]. 

#### 2.5.2. Lipid Peroxidation

Hernández and Almansa [38] assessed lipid peroxidation in terms of malondialdehyde (MDA) concentration. In a nutshell, 500 mg of fresh plant tissue were mixed for 20 min at 4 °C in a 1 mL 20% (*w/v*) trichloroacetic acid (TCA) and centrifuged at 16,000× *g*. The supernatants were mixed with an equivalent amount of 0.65 percent thiobarbituric acid in a test tube, and the 20% trichloroacetic acid solution was kept in an incubator at 96 °C for 30 min. Finally, the solution was chilled by keeping it in an ice bath. The absorbance of the supernatant was measured at 532 nm.

#### 2.5.3. Measurement of ROS Indicators

To assess hydrogen peroxide (H_2_O_2_) content, samples were extracted in 5% trichloroacetic acid (TCA) and centrifuged at 11,000× *g* for 15 min. The absorbance at 390 nm was measured after the supernatant was mixed with one mM KI and ten mM phosphate buffer (pH 7.0) [39]. Hydroxyl radical (OH) concentration was calculated following Halliwell et al. [40]. The reaction mixture consisted of deoxyribose, KH_2_PO_4_-KOH buffer (20 mM, pH 7.4), 100 μM ascorbate, 104 μM EDTA, 100 μM FeCl_3_, and 1 mM H_2_O_2_. After 1 h of incubation at 37 °C, the optical density was determined at 532 nm. The supernatant was also used to calculate superoxide anion (O_2_^−^) by mixing it with hydroxylamine hydrochloride and heating it at 25 °C for one hour, then mixing it with sulfanilamide and naphthylamine and heating it at 25 °C, then read at 530 nm [41].

### 2.6. Enzymatic Antioxidant Assays

Half a gram of fresh leaf was crushed in 10 mL of 50 mM KH_2_PO_4_ buffer (pH 7.8) and centrifuged for 15 min at 10,000× *g*. The extract’s protein content was then determined. A catalase activity test (CAT) was performed using H_2_O_2_ as a substrate and potassium phosphate as a buffer, as Aebi [42] reported, and at 240 nm, absorbance was measured. The Kono [43] technique was used to assess superoxide dismutase (SOD) activity using nitro blue tetrazolium (NPT) as the substrate and Na_2_CO_3_ as a buffer. Inhibition of NBT decrease was determined using 540 nm. The peroxidase (POX) activity analysis was carried out by Thomas et al. [44] using benzidine, and the absorbance was at 470 nm spectrophotometer. The activity of ascorbate peroxidase (APX) was tested by Nakano and Asada [45]. Five mM ascorbate, potassium phosphate buffer, enzyme extract, and 0.5 mM H_2_O_2_ were utilized in the reaction mixture. At 265 nm, the absorbance was measured. Glutathione reductase (GR) activity was measured at 340 nm after 1 min of NADPH oxidation, according to Jiang and Zhang [46]. Lipoxygenase (LOX) activity was assessed by reading absorbance at 234 nm [47]. The Jaworski [48] approach was used to measure nitrate reductase activity (NR). Fresh leaves were placed in vials with phosphate buffer, isopropanol, and KNO_3_, and incubated for 2 h at 30 °C. Naphthylethylene diamine hydrochloride, sulfanilamide, and solution were added after incubation and measured at 540 nm. The activity of carbonic anhydrase (CA) in the leaves was assessed using the Dwivedi and Randhawa [49] technique, in which the leaves were dried and transferred to the tube, along with bromothymol blue, 0.4 M NaHCO_3_, phosphate buffer, and ultimately, the methyl red indicator.

### 2.7. Determination of α-Tocopherol, Lignin, and Ethylene Content 

The α-tocopherol was assayed according to Kivçak and Mert [50]. First, in prechilled chloroform, fresh leaves were homogenized. Next, 1 mL of extract, 1 mL of ferric chloride, and then 1 mL of 2,2-dipyridyl reagent were mixed and shaken for 10 s. After adding ferric chloride, the absorbance was measured at 522 nm. Next, the lignin content was determined by Bruce and West [51]. The sample was applied with 30 mL of 7.5 mM of hydroxylamine, 900 mL of acetic acid, and 270 mL of 2 mM NaOH, and the lignin content was read at 280 nm. Finally, the amount of ethylene produced at the roots was determined, according to Sun et al. [52]. The root pieces were cut 2 cm from the apex of the root and put in 2 mL plastic vials containing 0.7 percent agar medium, where they were cultured in the dark at 25 °C. After incubation, one mL of gas was immediately fed into a gas chromatography system (GC 7890A, Agilent Technologies, Santa Clara, CA, USA) and a flame ionization detector.

### 2.8. Determination of Mineral (N, P, K) Content 

Dried powdered tissues were used for the estimation of nitrogen (N), potassium (K), and phosphorus (P). N was estimated using the micro-Kjeldahl apparatus (Ningbo Medical Instruments Co., Ningbo, China) following Bremner [53], P was determined following Sen Tran et al. [54], and K was determined using a flame photometer [55].

### 2.9. Determination of Metal Concentration, Accumulation, and Translocation in Tomato Plants

According to Tatiana et al. [56], the metal content of Cd and Ni in tomato plants was measured. The plants were carefully harvested, separated into shoots and roots, and dried for 48 h at 65 °C. To eliminate non-specifically bound Cd and Ni, the shoots and roots were cleaned with distilled water and 0.01 M EDTA (ethylenediaminetetraacetic acid). Furthermore, dried plant samples were digested in a ratio of 1:3:1 on a hot induction plate (HNO_3_:H_2_SO_4_:HClO_4_, *v*/*v*). After that, the samples were cooled. The samples were diluted again with distilled water to a final volume of 50 mL. An atomic absorption spectrophotometer calculates the amount of metal accumulated in the leaves and roots.

The biological accumulation factor (BCF) or the bioaccumulation factor measured the capacity of plants to collect components from the soil to the plant [57].

$$BCF=\frac{Total\;metal\;content\;in\;the\;shoots\;or\;roots\;}{The\;content\;of\;metal\;applied\;in\;soil.}$$

### 2.10. Gene Expression Analysis through Quantitative Real-Time (qRT-PCR)

According to the manufacturer’s instructions, total RNA was extracted using Trizol (Invitrogen, Life Technologies, Carlsbad, CA, USA). The approach suggested by Awasthi et al. [58] was used to synthesize cDNA. Primer3 software was used to build primers for qRT-PCR investigations [59] (Table 1). The ubiquitin gene was used as a housekeeping internal reference gene for normalization. Using the 2^−ΔΔCT^ technique, the relative expression level was calculated. 

### 2.11. Statistical Analysis

In the study, an entirely randomized design (CRD) was used, which comprised nine treatments and six replications. As a consequence, SPSS was used to conduct statistical analysis. Fisher’s two-way ANOVA with a 95% confidence level. The parametric distribution (normality) of the Levane test was used. The heat map displays the Pearson correlation and cluster analysis. It was computed to see whether there was a link between quantitative parameters [60]. GraphPad Prism 8 was used to make the graphs.

## 3. Results

### 3.1. Isolation, Characterization, and Biochemical Identification of Endophytic Bacteria 

A total of 63 bacterial isolates were previously isolated (tomato plant samples) in the plant virus and bacteriophage Lab of Botany and Micro. Dep., Fac. of Sci., Al-Azhar Univ. Cairo, Egypt. 

Bacterial isolates were generally screened for their ability to promote plant growth, which showed high diversity, with one or more effects, and 2 isolates were effective endophytic bacteria (Table 2). The amounts of IAA production were about 8.77 ± 0.34, 8.69 ± 0.40 μg mL^−1^, and the root colonization ability was around 6.54 ± 0.11, 6.49 ± 0.31 log_10_ CFU/g. Additionally, these isolates were able to solubilize phosphate by 10.23 ± 0.31, 7.03 ± 0.32 μg mL^−1^. All the 2 endophytic bacterial isolates exhibited a biofilm production behavior of about 1.38 ± 0.07, 1.21 ± 0.04 OD_570_.

Based on the morphological and biochemical characteristics of the selected endophytic bacterial isolates (Table 3), *Micrococcus luteus* isolates were Gram-positive. 

**Figure 1:**
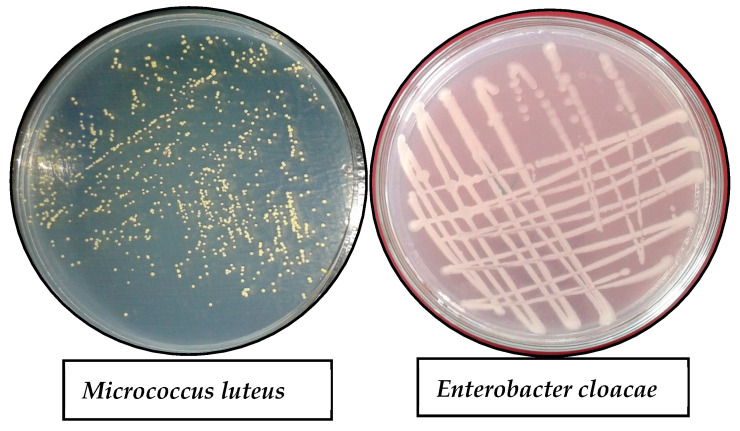
NA plates of two heavy metal tolerant strains, *Micrococcus luteus*, and *Enterobacter cloacae*.

Regarding heavy metal tolerance, among Gram-positive and Gram-negative endophytic bacterial isolates, only two isolates exhibited more resistance in this study (Table 3). One was Gram-positive (EBI-23) named *Micrococcus* spp., while the other was Gram-negative (EBI-45) *Enterobacter* spp. (Figure 1). 

The two bacteria demonstrated higher tolerance to Cd and Ni at concentrations of 3.125, 6.25, 12.5, 25, and 50 μM, while the growth of the two isolates was affected at 100 μM. Based on this screening, the tolerable behavior for both bacteria showed more tolerance against Ni than Cd heavy metals. Therefore, depending on these results, the two isolates might enhance heavy metal tolerance and plant growth (Table 4).

### 3.2. Molecular Identification of the 16S rDNA Gene for the Two Bacterial Isolates

Genomic DNA extracted from selected bacterial isolates was subjected to universal primer pair designation to amplify the 16S rDNA region. PCR amplification of the 16S rDNA gene produced the expected amplicons size of approximately 1520 bp. Additionally, partial nucleotide sequences of the PCR-amplified fragments of the 16S rDNA gene of 2 bacterial isolates were obtained to determine the relationship with other recommended bacterial isolates registered in GenBank. The nucleotide sequences were submitted in the GenBank under accession numbers, Enterobacter OM519328 and Micrococcus OM519327.

### 3.3. Bioinformatics Analysis of Isolates Enterobacter Cloacae and Micrococcus Luteus Nucleotide Sequence 

**Figure 2:**
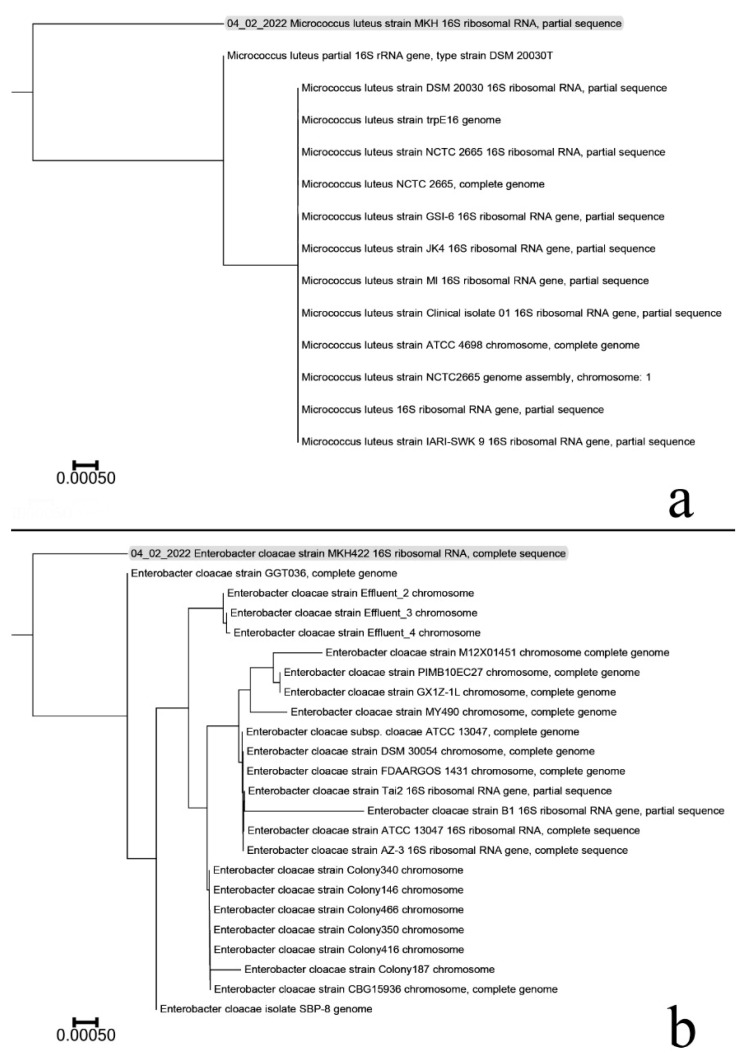
Based on 1000 replicates, the neighbor-joining tree of the 16S rDNA gene of the isolate *Enterobacter cloacae* (Accession no. OM519328) and 23 *Enterobacter cloacae* sequences (**a**). *Micrococcus luteus* (Accession number OM519327) and 13 *Micrococcus luteus* sequences (**b**).

Utilizing the NCBI n-BLAST search program at the National Center for Biotechnology Information (NCBI), the NCBI compared the partial nucleotide sequences of the *Enterobacter cloacae* 16S rDNA gene and *Micrococcus luteus* 16S rDNA gene with similar sequences retrieved from DNA databases. Multiple alignments were achieved using the ClustalW program with some minor manual adjustments using 23 reported *Enterobacter cloacae* and 13 reported *Micrococcus luteus* sequences in GenBank. A phylogenetic tree was generated based on the neighbor-joining method and with 1000 bootstrap repetitions (Figure 2a,b). It was reported that the two bacteria belonged to the genus *Enterobacter* and *Micrococcus* and were closely clustered with *Enterobacter cloacae* and *Micrococcus luteus*. The amplified 16S rDNA gene sequence isolating *Enterobacter cloacae* showed maximum homology (100%) with other isolates in GenBank. Furthermore, *Micrococcus luteus* showed maximum homology (%).

### 3.4. Variations in Growth Parameters

**Figure 3:**
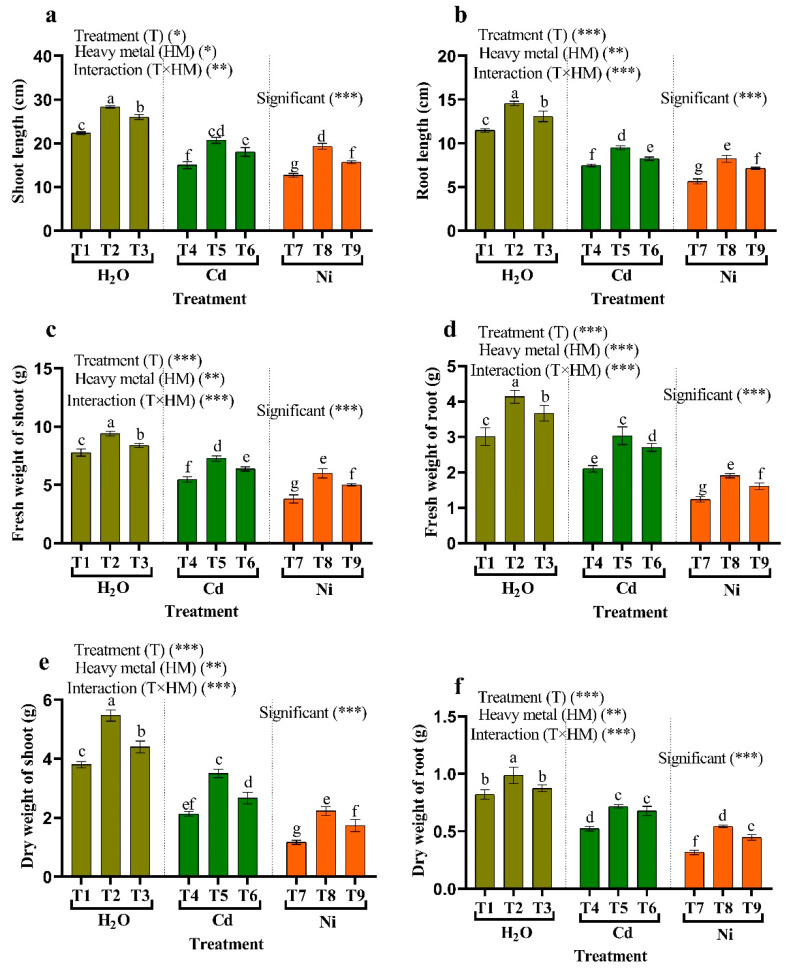
The influence of endophytic bacteria on (**a**) shoot length, (**b**) length of the root, (**c**) fresh weight of shoot, (**d**) dry weight of shoots, (**e**) fresh weight of the root, (**f**) dry weight of the root in tomato plants growing under HM stress. Fisher’s test at *p* < 0.05 reveals significant variations in means (±standard error), which are different letters (a–g) on the same bars. *, ** and *** imply significance levels of 0.01 and 0.05, respectively. T1: (Control with tap water), T2: *Micrococcus luteus*, T3: *Enterobacter cloacae*, T4: 50 μM Cd, T5: *Micrococcus luteus* + 50 µM Cd, T6*: Enterobacter cloacae* + 50 µM Cd T7: 50 µM Ni, T8: *Micrococcus luteus* + 50 µM Ni, T9*: Enterobacter cloacae* + 50 µM Ni.

Figure 3a–f illustrates some of the tomato plant’s growth characteristics, such as shoot, root length, shoot, and root fresh weight, and shoot and root dry weight when applied with heavy metals (HMs) and isolated bacteria such as *Micrococcus luteus* and *Enterobacter cloacae*. The application of 50 µM Cd or Ni to tomato plants declines the growth characteristics. Similarly, the length of the shoot was significantly reduced when the plants were irrigated with 50 µM of Cd or N, by approximately 23.83% and 43.28%, respectively, in contrast to the plants without stress. On the contrary, endophytic bacteria (*Micrococcus luteus* and *Enterobacter cloacae*) significantly improved growth characteristics in HMs-stressed plants compared to non-stressed plants. Plants treated with *Micrococcus luteus* showed the most pronounced rise. As a result, the length of the shoot has risen about 37.78% and 52.62% compared to the stress of the HM. Thus, treated tomato plants with 50 µM of Cd or Ni illustrate a decline in fresh and dry weight of shoots at about 29.61% and 51.08% at Cd stress, and 43.86% and 69.30% at Ni stress, respectively. However, *Micrococcus luteus* showed an increase in fresh and dry weight of the shoots under 32.92% and 57.89% at Cd stress, and 64.07% and 91.39% at Ni stress, respectively, under stress (Figure 3). Furthermore, fresh and dry weight of roots cultivated below 50 µM of Cd and Ni stress decreased by 30.00%, 58.90%, 36.9 5%, and 71.57%, respectively. On the contrary, significant increases in fresh and dry weight of roots of tomato plants under HM stress plants (50 µM of Cd or Ni) when treated by *Micrococcus luteus* (Figure 3).

### 3.5. Chlorophyll Content and Photosynthetic Characteristics

**Figure 4:**
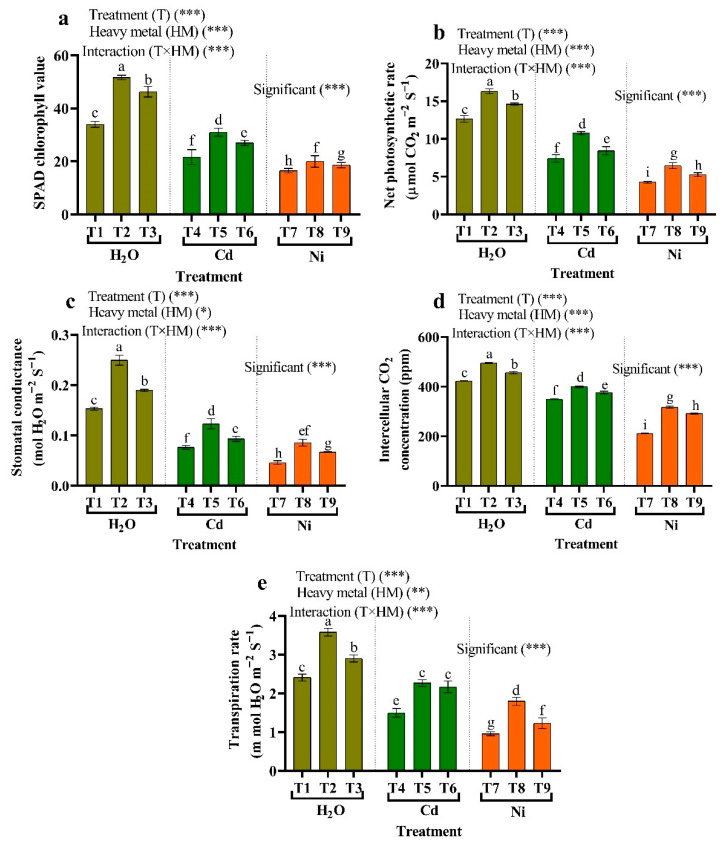
The influence of two different endophytic bacteria on (**a**) SPAD chlorophyll values, (**b**) net photosynthetic rate (PN), (**c**) stomatal conductance (gs), (**d**) intercellular CO_2_ concentration (Ci), and (**e**) transpiration rate (E) in tomato plants growing under HM stress. Fisher’s test at *p* < 0.05 reveals significant variations in means (±standard error), which are different letters (a–i) on the same bars. Thus, *, ** and *** imply significance levels of 0.01 and 0.05, respectively. T1: (Control with tap water), T2: *Micrococcus luteus*, T3: *Enterobacter cloacae*, T4: 50 μM Cd, T5: *Micrococcus luteus* + 50 µM Cd, T6*: Enterobacter cloacae* + 50 µM Cd T7: 50 µM Ni, T8: *Micrococcus luteus* + 50 µM Ni, T9*: Enterobacter cloacae* + 50 µM Ni.

Figure 4 shows the SPAD chlorophyll values, and photosynthetic properties (intercellular CO_2_ concentration (Ci), net photosynthetic rate (PN), stomatal conductance (gs), and transpiration rate (E)) decreased in leaves with HMs treatment comparison to control plants. In contrast, the contents of SPAD chlorophyll and the photosynthetic properties were raised in tomato plants by applying endophytic bacteria (*Micrococcus luteus* and *Enterobacter cloacae*). Endophytic bacteria mitigated the adverse effects of HMs (*Micrococcus luteus*), where the chlorophyll content of SPAD increased by 34.05% and 21.21%, PN increased by 44.85% and 51.56%, gs increased by 60.82% and 86.96%, Ci increased by 14.39% and 49.84%, and E increased by 51.11% and 88.15%, compared to metal stress (Cd and Ni) (Figure 4).

### 3.6. Osmolytes (Proline and Phenol)

**Figure 5:**
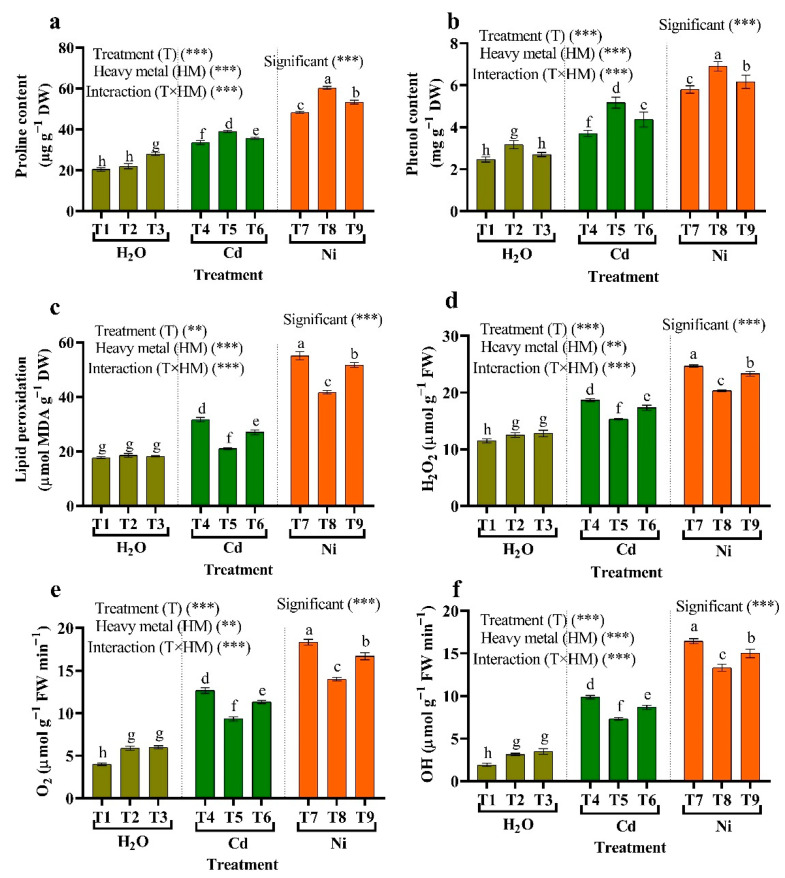
The influence of two different endophytic bacteria on (**a**) proline, (**b**) phenol, (**c**) lipid peroxidation (MDA), (**d**) hydrogen peroxide (H_2_O_2_), (**e**) hydroxyl radicals (OH), and (**f**) superoxide anion (O_2_) in tomato plants growing under HM stress. Fisher’s test at *p* < 0.05 reveals significant variations in means (±standard error) of different letters (a–h) on the same bars. Thus, ** and *** imply significance levels of 0.01 and 0.05, respectively. T1: (Control with tap water), T2: *Micrococcus luteus*, T3: *Enterobacter cloacae*, T4: 50 μM Cd, T5: *Micrococcus luteus* + 50 µM Cd, T6*: Enterobacter cloacae* + 50 µM Cd T7: 50 µM Ni, T8: *Micrococcus luteus* + 50 µM Ni, T9*: Enterobacter cloacae* + 50 µM Ni.

The osmolytes content (proline and phenol) increased significantly under HMs compared with non-stressed plants (Figure 5). However, at 50 µM Ni, the maximum content of proline and phenol content was recorded. In addition, treatment with *Micrococcus luteus* and *Enterobacter cloacae* under HMs conditions produced a considerable rise in proline and phenol content compared to plants stressed with HMs. Under 50 µM Cd and Ni, significant increases in proline and phenol content were detected when tomato plants were applied with *Micrococcus luteus* compared to stressed plants and extended to control plants well. 

### 3.7. Lipid Peroxidation Content and ROS Production 

As a consequence of these results, some critical observations may be made on the development of MDA as an indicator of lipid peroxidation and ROS production (H_2_O_2_, OH, and O_2_) in tomato leaves treated with *Micrococcus luteus*. *Enterobacter cloacae* in the presence or absence of HMs stress are presented as shown in Figure 5. Our results show that allowing tomato plants to grow under stress derived from elevated Cd and Ni caused a significant rise in MDA, H_2_O_2_, OH, and O_2_ content. Applying *Micrococcus luteus* and *Enterobacter cloacae* proved its substantial capability to alleviate HMs-stress by reducing the MDA, H_2_O_2_, OH, and O_2_ content. When using a blend of *Micrococcus luteus*, the lower MDA, H_2_O_2_, OH, and O_2_ contents were recorded in all stressed plants.

### 3.8. Changes in Antioxidant Enzymes

**Figure 6:**
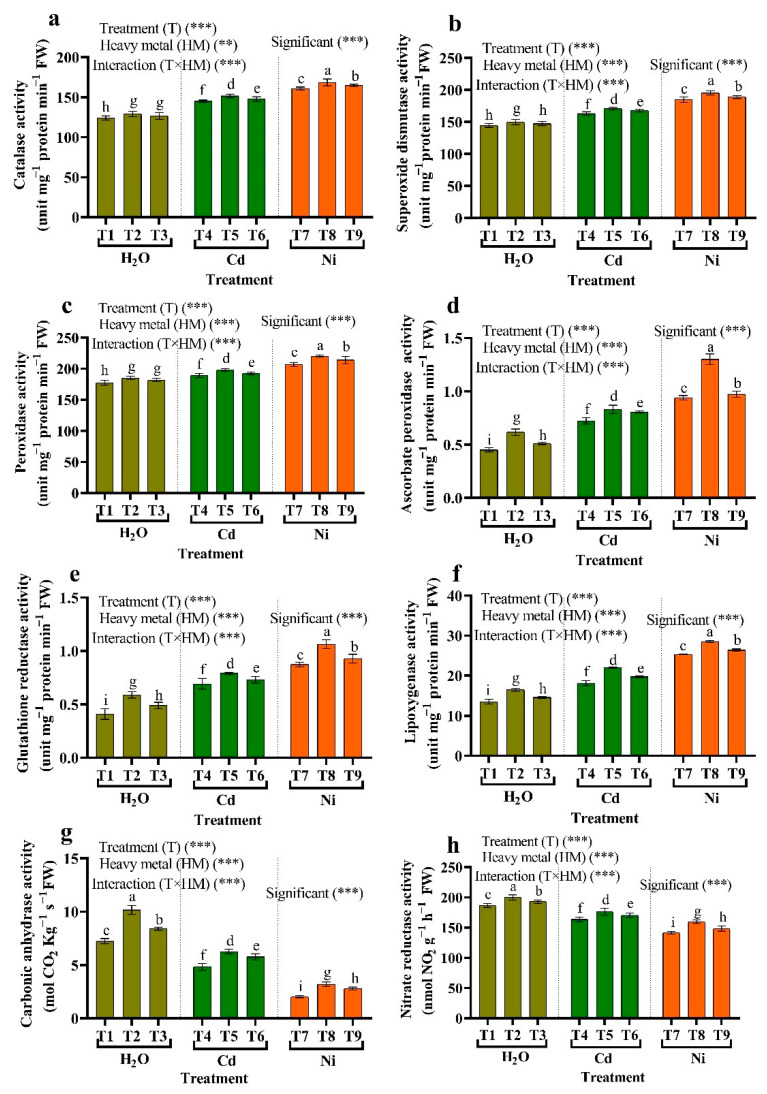
The influence of two endophytic bacteria on (**a**) catalase, (**b**) superoxide dismutase, (**c**) peroxidase, (**d**) ascorbate peroxidase, (**e**) glutathione reductase, (**f**) lipoxygenase, (**g**) carbonic anhydrase and (**h**) nitrate reductase activity in tomato plants growing under HM stress. Fisher’s test at *p* < 0.05 reveals significant variations in means (±standard error) of different letters (a–i) on the same bars. Thus, ** and *** imply significance levels of 0.01 and 0.05, respectively. T1: (Control with tap water), T2: *Micrococcus luteus*, T3: *Enterobacter cloacae*, T4: 50 μM Cd, T5: *Micrococcus luteus* + 50 µM Cd, T6*: Enterobacter cloacae* + 50 µM Cd T7: 50 µM Ni, T8: *Micrococcus luteus* + 50 µM Ni, T9*: Enterobacter cloacae* + 50 µM Ni.

When HM-stressed plants were compared to non-stressed plants, CAT, APX, POX, GR, SOD, and LOX activities in the tomato plant leaves were significantly raised (Figure 6). However, the activities of CAT, POX, SOD, APX, GR, LOX, NR, and CA activities increased significantly in HMs-stressed plants due to the treatments (i.e., *Micrococcus luteus*, *Enterobacter cloacae*) compared to stressed plants. NR and CA activities, in contrast, were considerably reduced in HM-stressed plants than in non-stressed plants. Since endophytic bacteria mitigated the adverse effects of HMs with (*Micrococcus luteus*), where the CAT content increased by 4.60% and 4.76%, SOD increased by 4.69% and 5.59%, POX increased by 4.58% and 6.61%, APX increased by 14.75% and 38.30%, GR increased by 13.94% and 21.75%, LOX increased by 21.33% and 21.63%, CA increased by 29.66% and 57.38%, and NR increased by 7.74% and 12.97% compared to metal stress (Cd and Ni) (Figure 6).

### 3.9. α-Tocopherol, Lignin, Ethylene, and Mineral Content

**Figure 7:**
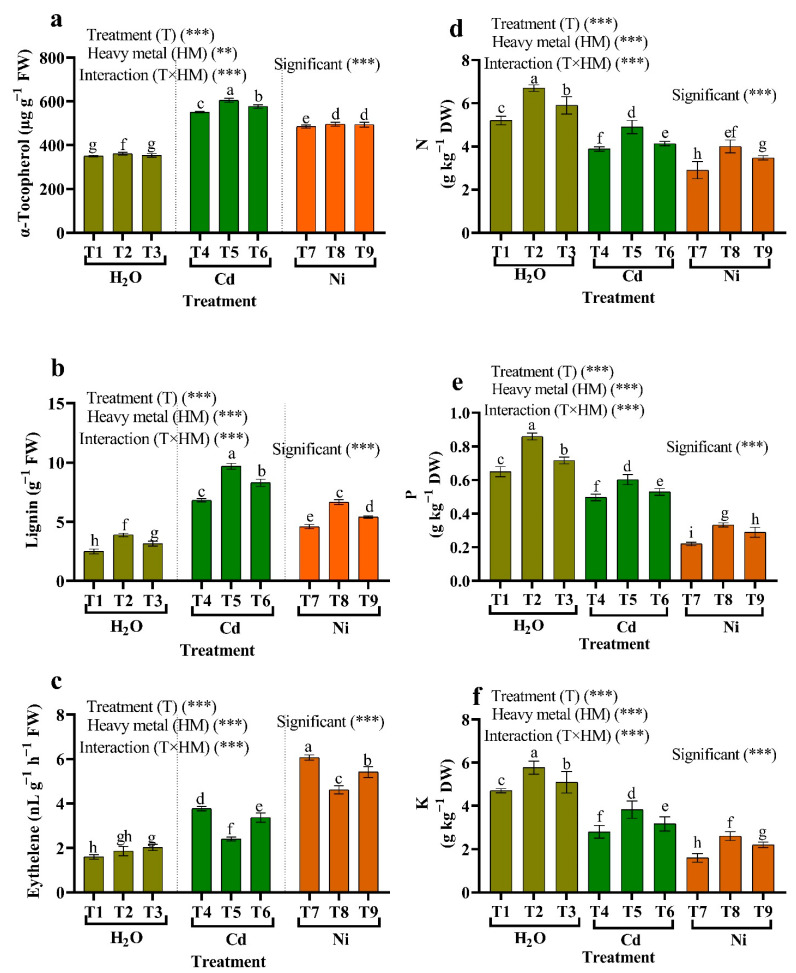
The influence of two endophytic bacteria on (**a**) α-Tocopherol, (**b**) lignin, (**c**) ethylene, (**d**) nitrogen (N), (**e**) phosphor (P), and (**f**) potassium (K) content in tomato plants growing under HM stress. Fishers test at *p* < 0.05 reveals significant variations in means (±standard error) of different letters (a–i) on the same bars Thus, ** and *** imply significance levels of 0.01 and 0.05, respectively. T1: (Control with tap water), T2: *Micrococcus luteus*, T3: *Enterobacter cloacae*, T4: 50 μM Cd, T5: *Micrococcus luteus* + 50 µM Cd, T6*: Enterobacter cloacae* + 50 µM Cd T7: 50 µM Ni, T8: *Micrococcus luteus* + 50 µM Ni, T9*: Enterobacter cloacae* + 50 µM Ni.

In HM plants, α-Tocopherol (57.73% and 38.44%), lignin (173.33%, 84.00%) and ethylene content (134.40% and 279.17%) increased as compared to non-stress plant (Figure 7). α-Tocopherol and lignin content was increased in *Micrococcus luteus*-treated samples compared to Cd stress. The most potent increase in α-Tocopherol and lignin content was observed in the binary treatment of *Micrococcus luteus* compared to Ni stress. Treatment with *Micrococcus luteus* and *Enterobacter cloacae* decreased the ethylene content in Cd stress and Ni stress (Figure 7). Our results show that allowing tomato plants to grow under HM stress derived from elevated Cd and Ni caused a significant decrease in the content of N, P, and K. Applying *Micrococcus luteus* and *Enterobacter cloacae* proved their substantial capability to alleviate HM-stress by increasing the N, P, and K content.

### 3.10. Effect of Endophytic Bacteria on Cd, Ni Concentration, and BCF of Cd and Ni 

**Figure 8:**
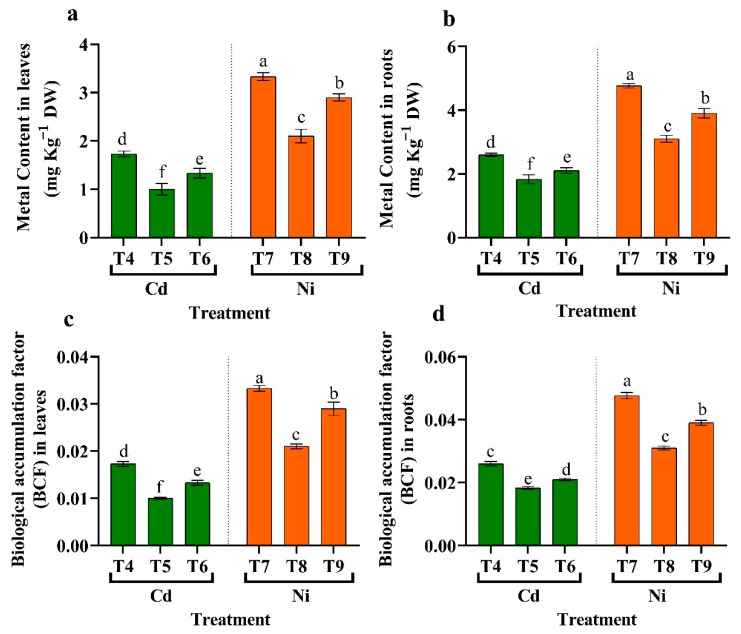
The influence of two endophytic bacteria on Cd, and Ni concentration in (**a**) leaves, (**b**) roots and BCF of Cd and Ni in (**c**) leaves, and (**d**) roots of tomato plants growing under HM stress. Fisher’s test at *p* < 0.05 reveals significant variations in means (±standard error) of different letters (a–f) on the same bars. T4: 50 μM Cd, T5: *Micrococcus luteus* + 50 µM Cd, T6*: Enterobacter cloacae* + 50 µM Cd T7: 50 µM Ni, T8: *Micrococcus luteus* + 50 µM Ni, T9*: Enterobacter cloacae* + 50 µM Ni.

The effect of endophytic bacteria *Micrococcus luteus* and *Enterobacter cloacae* treatment on tomato seeds was evaluated by detecting Cd and Ni accumulation and translocation in shoots and roots. Under stress conditions, plants treated with *Micrococcus luteus* and *Enterobacter cloacae* had significantly reduced levels of Cd and Ni than non-inoculated plants (Figure 8). Furthermore, treatment with *Micrococcus luteus* significantly reduced the content of Cd (42.31%, 29.49%) and Ni (37.00%, 34.97%) in the shoots and roots than non-treated stress without treatment (Figure 8). Furthermore, when endophytic *Micrococcus luteus* and *Enterobacter cloacae* were used compared to HMs only, BCF of Cd and Ni from water to roots and shoots were highly reduced. Moreover, the treatment of *Micrococcus luteus and Enterobacter cloacae* revealed a significantly reduced amount of BCF under Cd and Ni stress than HMs stress, respectively.

### 3.11. Antioxidant Enzyme Gene Expression

**Figure 9:**
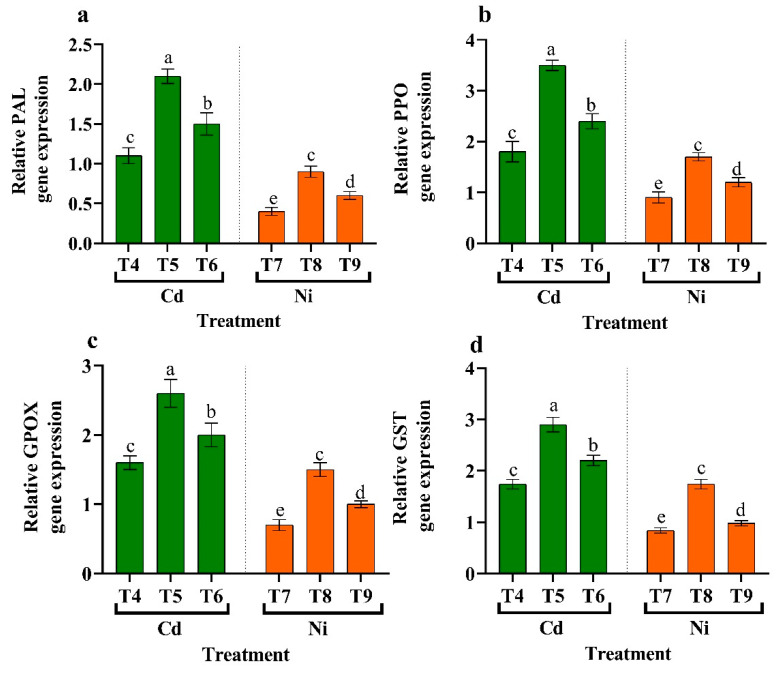
The influence of two endophytic bacteria on antioxidant enzyme gene expression ((**a**) PAL gene, (**b**) PPO gene, (**c**) GPOX gene, and (**d**) GST gene) in tomato plants growing under HM stress. Fisher’s test at *p* < 0.05 reveals significant variations in means (±standard error) of different letters (a–e) on the same bars. T4: 50 μM Cd, T5: *Micrococcus luteus* + 50 µM Cd, T6*: Enterobacter cloacae* + 50 µM Cd T7: 50 µM Ni, T8: *Micrococcus luteus* + 50 µM Ni, T9*: Enterobacter cloacae* + 50 µM Ni.

Quantitative real-time PCR was used to examine the expression of HM-related genes relative to PAL, PPO, GPOX, and GST in tomato leaves under stress and endophytic bacteria application (Figure 9). With the application of endophytic bacteria under HMs-stress circumstances, the relative expression levels of the PAL, PPO, GPOX, and GST genes significantly increased at the transcript level. Since endophytic bacteria mitigated the adverse effects of HMs with (*Micrococcus luteus*), where the PAL gene content increased by 90.91% and 125.00%, PPO gene increased by 94.44% and 33.33%, GPOX gene increased by 62.50% and 114.29%, and GST gene increased by 66.67% and 107.14% compared to metal stress (Cd and Ni) (Figure 9).

### 3.12. Correlation Analysis and Principal Component Analysis (PCA)

**Figure 10:**
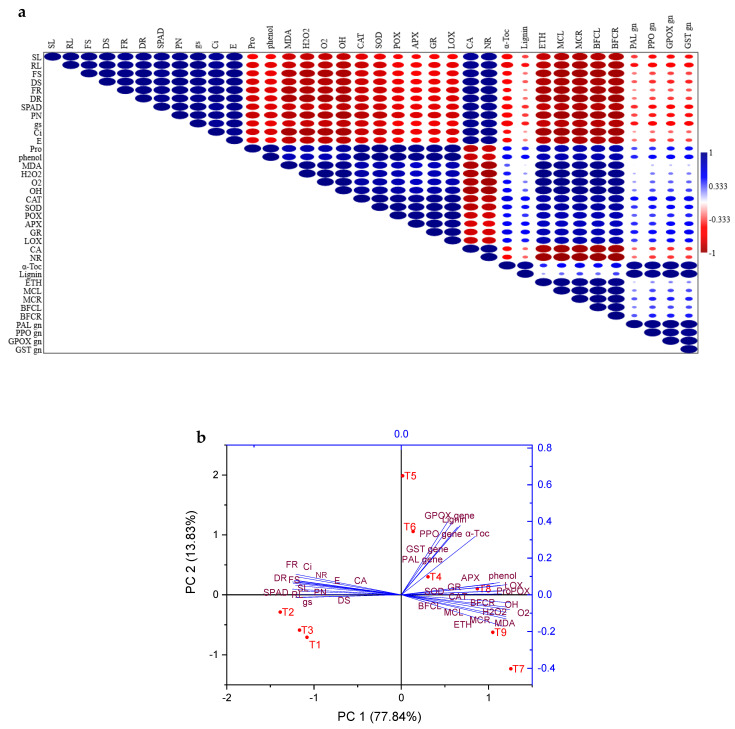
(**a**) The heat map confirms the association between quantitative statistical parameters based on the mean values of different parameters reported in this research. (**b**) Using principal component analysis (PCA) to analyze the correlations between treatment variables in tomato plants. Shoot length (SL), root length (RL), F W. of the shoot (FS), D W. of the shoot (DS), F W. of root (FR), D W. of the root (DR), SPAD chlorophyll value (SPAD), stomatal conductance (gs), net photosynthetic rate (PN), transpiration rate (E), internal CO_2_ concentration (Ci), proline (Pro), phenol, MDA, H_2_O_2_, OH, O_2_, CAT, POX, SOD, GR, APX, LOX, carbonic anhydrase (CA), nitrate reductase activity (NR), α-Tocopherol (α-Toc), lignin, ethylene, metal concentration in leaves (MCL), metal concentration in roots (MCR), BFC leaves (BFCL), BFC roots (BFCR), PAL gene (PAL gn), PPO gene (PPO gn), GPOX gene (GPOX gn), and GST gene (GST gn).

The correlation analysis under HMs (Cd and Ni) conditions and endophytic bacteria (*Micrococcus luteus* and *Enterobacter cloacae)* revealed that growth parameters, photosynthetic characteristics, proline, phenol content, lipid peroxidation, ROS indicators, antioxidant activity, α-tocopherol, lignin, ethylene content, metal concentration, accumulation, translocation, and gene expression had a significant correlation (Figure 10). Furthermore, there was a significant and positive correlation between the morphological characteristics and biochemical characteristics (SPAD, PN, gs, Ci, E, CA, and NR). On the other hand, there was a negative and significant correlation between morphological characteristics and biochemical (proline, phenol, MDA, H_2_O_2_, OH, O_2_, CAT, POX, SOD, APX, LOX, GR, α-Tocopherol, lignin, ethylene, metal concentration in leaves, roots, BFC leaves and roots, PAL gene, PPO gene, GPOX gene, and GST gene) parameters. The multifactorial effects of our treatments on all variables of tomato plant stress under HM and non-HM stress were clarified using principal component analysis (PCA). To explain the total variance of the examined attributes using the cross-validation approach, two key components were required. A total of 91.67% of the data variability—77.84% and 13.83% for PC1 and PC2, respectively—was represented by the two components (PC1 and PC2) that were recovered from the covariance matrix’s eigenvalues (Figure 10).

## 4. Discussion

It has been found that plant growth-promoting rhizobacteria (PGPR) are important in helping plants handle abiotic stresses and maintain productivity [61]. Biologically beneficial soil bacteria can live symbiotically with plants or endophytically within their host plants in the rhizosphere. They aid plant growth by producing enzymes, phytohormones, solubilizing minerals, biological nitrogen fixation, mineralizing organic phosphate, generating amino acids, and enhancing the bioavailability of nutrients in the rhizosphere via modifying converting and permeability of nutrients [62]. Plants can cope with abiotic stress by lowering ethylene levels, producing and accumulating solutes such as glycine betaine proline, and lowering ROS generation using PGPR [63]. Therefore, the use of PGPR could be considered a key strategy for sustainable agriculture in reducing plant oxidative and osmotic stress [64].

Based on its ability to grow in Cd and Ni and its ability to promote plant growth, HM-resistant endophytic PGPR was selected for further investigation in the present research. *Micrococcus luteus* and *Enterobacter cloacae* both demonstrated a high ability to solubilize phosphate. Plants require phosphorus, second only to nitrogen as one of the most important nutrients. Soil contains most phosphorus in the form of insoluble phosphates, which plants are unable to utilize [65]. Similar to our finding, *Pseudomonas pseudoalcaligenes* and *Bacillus pumilus* were capable of dissolving phosphorus by generating gibberellins and auxins [66]. In addition, there are low molecular weight compounds called siderophores that scavenge iron, which is prevalent in the environment and makes it available to microorganisms, making it available to plants through roots, thereby promoting plant growth [67].

Plants differ in their ability to absorb Cd and Ni from polluted soil and transfer it from roots to shoots. Plant development is slowed by Cd and Ni stress, which is frequent in agriculture, and Cd and Ni accumulation on non-edible plant components [68]. Moreover, it restricts the distribution and absorption of mineral nutrients [69]. Cd and Ni stress has been connected to the long-term persistence of HM in soil and water, which has a considerable detrimental impact on agricultural performance and human health when plants are consumed [70]. The build-up of HMs in agriculture is widely documented to have significant harmful effects on plant systems, including impaired plant growth [71], leaf chlorosis [72], and undesired enzyme activation and inhibition [73]. Cd and Ni stress additionally impacts metabolic activities such as cell elongation and meristematic activity associated with greater respiration rates due to raised energy needs [74]. Furthermore, Cd and Ni stress increase ROS generation, producing oxidative plant tolerance [75]. ROS negatively impacts DNA, chlorophyll, proteins, and membrane function. Plants activate their antioxidant systems to repair and minimize the damage produced by ROS [76], which include many low-weight non-enzymatic molecules (e.g., GSH, proline, carotenoids, and AsA) and enzymatic substances (e.g., POX, CAT, SOD, GR, and APX) [67]. In most cases, these powerful antioxidant defense mechanisms are insufficient to help plants deal with stress [77].

Tomato plants were grown in Cd and Ni-soil without or with bacteria (*Micrococcus luteus* and *Enterobacter cloacae*) during various stages of plant growth in the present research. Data revealed a significantly reduced plant growth under Cd and Ni stress in the absence of *Micrococcus luteus* and *Enterobacter cloacae*. 

These findings are consistent with previous research revealing Cd and Ni toxicity in various plant species [67,78]. This halt in tomato development could be attributed to the adverse effects of Cd and Ni on plant growth, mineral accumulation, and physiology [34]. Our findings revealed that seed microbial inoculation positively impacted plant development characteristics. Microbes have a variety of impacts on improving plant development in adverse settings [13]. Microbes may reduce metal build-up in plants by altering the metal species in the soil, resulting in enhanced plant growth and biomass. Under experimental circumstances, the addition of *Micrococcus luteus* and *Enterobacter cloacae* improved the Cd and Ni-stressed tomato growth. In faba bean, endophytic bacteria have been shown to have beneficial benefits when exposed to metals [67], rice [79], and sesame [80]. Endophytic bacteria stimulate plant growth and development under various environmental stresses [63,81], which could be due to endophytic bacteria, especially *Micrococcus luteus-mediated* improvement of plant mineral nutrients.

Plants’ total chlorophyll levels are key stress indicators, although the generation of chlorophyll content and photosynthetic characteristics in leaves be reduced under various environmental pressures [12]. Stress with Cd and Ni significantly decreased the chlorophyll content and photosynthetic characteristics in tomato leaves. Under Cd and Ni toxicity, reduced synthesis of these photosynthetic pigments showed oxidative stress in leaves [12,82]. The increased Cd and Ni concentrations in tomato leaves may have increased oxidative stress, resulting in decreased chlorophylls exposed to HM. Under Cd and Ni stress, microbial treatment increased chlorophyll concentration and photosynthetic characteristics, and treatment with *Micrococcus luteus* increased these pigments even more. According to the research, microbes had a favorable influence on the chlorophyll content of plants cultivated under stressful circumstances [83]. Microbial fortification is engaged in repairing chlorophyll structures and higher photochemical efficiency in plants under Cd stress [84]. According to the current research, the enhancement of chlorophyll content in plants growing under Cd and Ni stress might be related to decreased Cd, Ni content, and oxidative stress in tomato plant leave. Kamran et al. [85] also found that Ni stress reduced chlorophyll content in wheat plants and that sewage sludge flooded soils reduced chlorophyll content in *Eruca sativa*. On the other hand, PGPR inoculation restored the decrease of chlorophyll content by increasing the defense mechanism via the formation of various zymes [13]. The activity of protochlorophyllide reductase and aminolevulinic acid dehydratase enzymes, which are involved in the manufacture of chlorophyll content in combination with critical plant nutrients, may have been lowered by Ni toxicity [86]. Furthermore, *Micrococcus luteus* and *Enterobacter cloacae* strains have the ability to produce siderophores (Table 1), which are capable of chelating Fe and transporting it into the cell, thereby increasing iron concentrations and enhancing Fe transport and bioavailability, which is linked to increased chlorophyll synthesis in plants [87].

Proline is an important biomarker for predicting plant resistance to environmental stress. It helps plants avoid environmental stress by lowering ROS levels, stabilizing stress-related enzymes, and buffering cellular redox [17]. In this work, stressed tomato plants with Cd and Ni with or without inoculation with *Micrococcus luteus* and *Enterobacter cloacae* collected much more proline and phenol than non-stressed plants. In reaction to HMs, Brilli et al. [88] discovered that plants with high levels of proline and phenol in their tissues perform the role of a radicals-free scavenger, an osmolyte, and cellular redox protective agent. In wheat plants, an improvement in proline content was observed in reaction to Cd toxicity [89]. By functioning as a singlet oxygen scavenger, a radical hydroxyl scavenger, a lipid peroxidizing inhibitor, and proline may reduce the harmful effects of ROS [90]. Our findings demonstrated that each treatment’s proline levels in PGPR inoculated and non-inoculated plants were considerably different. In Cd and Ni polluted soil, the proline content of non-inoculated tomato plants increases more than that of plants grown in control soil without metal treatment. Our findings agree with Pramanik et al. [91], who found that proline rose considerably when rice seed was infected with PGPRs under Cd stress. In plant cells, proline is vital for protein chemistry, membrane strength, buffering cellular redox potential, and scavenging free radicals. Furthermore, in HM, high proline levels are caused by inhibition of degradation pathways or de novo synthesis [92]. 

The findings demonstrate that MDA H_2_O_2_, OH, and O_2_ levels in Cd and Ni stressed tomato shoots were considerably higher than in unstressed plants. ROS causes metabolic disorders and cell death by oxidizing DNA, proteins, and lipids [93]. The MDA concentration rose considerably in lettuce under the stress of Cd [12] and in maize plants under Ni stress [10]. Metal toxicity causes a reduction in enzyme activity in plants, which could explain the rise in malondialdehyde levels [94]. Unlike non-inoculated plants, inoculation with *Micrococcus luteus* and *Enterobacter cloacae* reduced MDA H_2_O_2_, OH, and O_2_ in Cd- and Ni-stressed plants. In comparison to non-inoculated plants, Cd-induced membrane damage and oxidative stress in plants treated with PGR may be minimized by lowering MDA H_2_O_2_, OH, and O_2_ [95].

The strains of *Micrococcus luteus* and *Enterobacter cloacae* can activate and regulate the activities of different antioxidant enzymes POX, CAT, APX, SOD, LOX, CA, GR, and NR in the tomato plant to help the plant survive under HM stress. Excess ROS were produced in plants as a result of disturbance of cell homeostasis caused by environmental stress, such as HM stress. Oxidative stress occurs when the quantity of ROS created exceeds the cell’s immunological defenses, resulting in decreased enzyme function, MDA, nucleic acid destruction, protein oxidation, activation of the main apoptotic pathway, and cell death [76]. As a result, antioxidant enzymes may help avoid harm by eliminating excess ROS created during HM stress. Many research findings have shown that overexpression of various antioxidant enzymes can enhance stress resistance. SOD is an important antioxidant enzyme that protects mitochondria, chloroplasts, peroxisomes, cell walls, endoplasmic reticulum, plasma membranes, and apoplast from abiotic and biotic stress because it is the first and most effective line of defense against ROS in such locations [96]. SOD activity has risen in response to Cd stress in chickpeas [97]. In the Haber–Weiss process, SOD is a critical antioxidant enzyme that scavenges O_2_, OH, and H_2_O_2_ [96]. Thus, the tomato plant has been protected against free radical-induced membrane dysfunction by rising the action of the enzymes CAT, APX, GR, SOD, POX, and LOX. Significantly raised POX activity in tomato plants infected with *Micrococcus luteus* and *Enterobacter cloacae* may be related to an increased response of Cd and Ni to stress because of better lignin and other antioxidant compounds to reduce the production [12]. POX activity in maize increased under Cd stress, according to AbdElgawad et al. [94]. Elevated CAT activity in tomato leaves grown on HMs soil may be linked to low H_2_O_2_ levels [98].

Consequently, CAT is essential during HM and eliminates H_2_O_2_ to protect the organs and membrane [41]. According to Hashem et al. [99], PGPR inoculation of okra plants increased antioxidant-coding genes, contributing to greater tolerance to stress of HM. Sofy et al. [13] also showed increased antioxidant systems as a result of PGPR. Furthermore, stress-resistant plants have been shown to maintain and accumulate Redox components [100]. Plant interactions with *Micrococcus luteus* and *Enterobacter cloacae* have additionally increased the production of redox components, such as reduced glutathione; they function as electron donors in enzyme-catalyzed antioxidant processes. Glutathione reductase (GR) is an important component of the ROS scavenging pathway, which contains the ascorbate-glutathione cycle [96]. The enzymatic defense antioxidant system is one mechanism that plays a critical function in preventing and stabilizing oxidative damage. The antioxidant defense system relies on ROS and/or RNS quenching enzymes like POX, CAT, APX, SOD, LOX, CA, GR, and NR [17]. Different abiotic stress promotes the formation of RNS, including NO and NO_2_ radicals and non-radical such as N_2_O_4_, HNO_2_, NO, and ONOO [101]. Oxidative stress degrades cellular organelle components like proteins, lipids, and nucleic acids, interfering with normal membrane activities and cell metabolism, resulting in lipid peroxidation and cell death [102]. As a result, RNS output management is required to minimize harmful RNS consequences and ensure that their signaling functions are adequately executed [103]. Plants have developed many defensive mechanisms to coordinate the generation and removal of RNS to avoid oxidative damage and signal activity [104]. Secondary metabolites (α-Tocopherol and lignin) were raised in plants exposed to Cd and Ni and treated with endophytic bacteria. The findings are consistent with Mishra and Sangwan [105], who found that phenolic content rose in Cd-applied with *Erica andevalensis*. The location of adsorbed and neutralized, singlet oxygen quenching or degrading peroxides through antioxidants are some of the biological functions of phenolic compounds [106]. 

Under HM stress, mineral levels such as N, P, and K in tomato plants dramatically decrease. Inoculation with *Micrococcus luteus* and *Enterobacter cloacae* strains, alone or in combination, stimulated the mineral contents of HM stressed plants. Abu-Shahba et al. [12] discovered that nutrient absorption was reduced during HM stress owing to a decrease in root length, a restriction in hydraulic conductivity, a decrease in root branching, and an increase in root thickness. PGPR may directly increase nutrient availability in the root system and/or activate ion transport pathways in the root by increasing root porosity, root area, and mineral nutrient absorption [107]. The most important mineral nutrients for plants are phosphorus, potassium, and nitrogen, which are required for amino acid formation and protein activation and are promoted by bacteria. Bacteria can improve N_2_ fixation, controlled by the nif gene and other basic genes; they can also improve plant growth, yield, soil nitrogen retention, and soil properties [108]. Photosynthesis, energy conservation, and carbon metabolism all need phosphate as a structural and signaling molecule [109]. As the cell’s principal osmoticum, potassium regulates cell expansion, plasma membrane potential and transport, pH value, and a variety of other catalytic activities [96]. Potassium, nitrogen, and phosphorus deficiencies cause reduced plant development, turgor loss, greater vulnerability to HM stress and infections, as well as necrosis and chlorosis [110,111]. Plants connected to the PGPR gene have developed a variety of adaptation methods to deal with variations in nutrient availability, including changes in ion transporter expression, raised root growth to explore more soil volume, and increased soil acidity to absorb more mineral nutrients. Only microorganisms can mineralize and solubilize phosphate and potassium in their organic or insoluble forms [112]. The fact that a specific PGPB generates ACC-deaminase, an enzyme that promotes the absorption of essential nutrients such as N, K, and P, and hence enhances plant growth under abiotic stress, might potentially explain the growth promotion [113]. 

Several genes encoding antioxidant enzymes were expressed in HM tomato leaves and endophytic bacteria (i.e., *Micrococcus luteus* and *Enterobacter cloacae*) under HMs conditions and compared using quantitative methods RT-PCR (Figure 9). PAL, PPO, GPOT, and GST transcript levels rose considerably with increasing HM-stressed + *Micrococcus luteus* tomato leaves, followed by *Enterobacter cloacae* treated + HM-stressed tomato leaves, compared to the HMs control. The functions of various plant peroxiredoxins and their isoforms have not been well studied. Nonetheless, all enzymes are required to detoxify alkyl hydroperoxide in various plant components [114]. In HM-stressed tomato leaves treated with endophytic bacteria, the expression of antioxidant enzymes was increased.

## 5. Conclusions 

**Figure 11:**
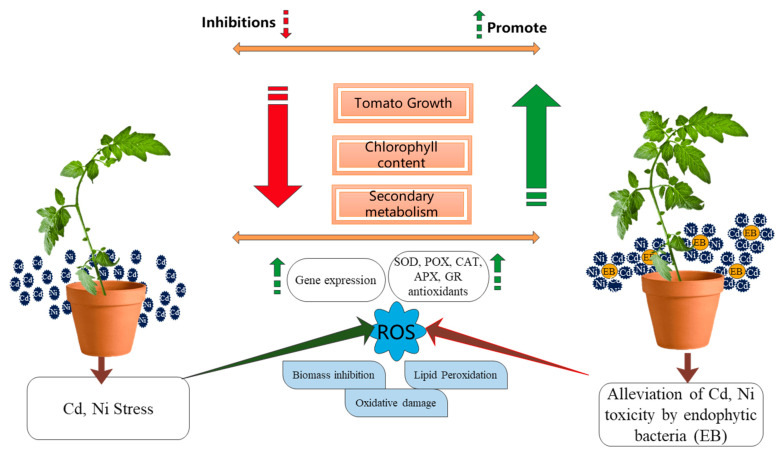
Mechanism of endophytic bacteria to promote plant growth and metabolites.

Finally, endophytic bacteria such as *Micrococcus luteus* and *Enterobacter cloacae* may boost plant biomass and growth and resist the harmful impact of metal emissions. This is due to their capacity to release active auxin, siderophores, ethylene, ACC deaminase, and secondary metabolites, which may also convert HMs into stable complexes. This might also be ascribed to a lower amount of metal within the roots, the shoots of tomato plants, and the transit of metal from soil to shoots. Thus, it was observed that treatment with *Micrococcus luteus* and *Enterobacter* resulted in the alleviation of Cd and Ni stress, as revealed by: (1) improved photosynthetic pigments, mineral nutrients, gene expression (PAL, PPO, GPOX, and GST), and reduced Cd accumulation; (2) decreased levels of ROS (3) modulation of total antioxidant enzyme activities, and reduced lipid peroxidation. In conclusion, *Micrococcus luteus* outperformed *Enterobacter cloacae* in the biosorption, phytoremediation, and bioaccumulation of Cd and Ni stress (Figure 11).

## Figures and Tables

**Table 1:** Forward and reverse primers sequence for PAL, PPO, GPOX, GST, and Ubiquitin genes.

	Primer Sequence (5′–3′)
Phenylalanine ammonia-lyase 5 (PAL)	F: CGGTGAGGAGATTGATAA R: TTAGCAGATTGGAATAGGA
Polyphenol oxidase (PPO)	F: TACTACTACAACGCTCAA R: AACCAAGAAGAACATTCC
Glutathione peroxidase (GPOX)	F: GAGATAATATTCAGTGGAATTTCGCTAA R: GTTGAGGGCTCAACCTT
Glutathione-S-transferase (GST)	F: CATTTGTTATGAATTTATTGAGCAAGAT R: TAAGTGGCCATGTTTCTTCAATATAC
Ubiquitin	F: GAGGAATGCAGATCTTCGTG R: TCCTTGTCCTGGATCTTAGC

**Table 2:** Patterns of the most potent plant growth-promoting endophytic bacterial isolate. ++ +++ (positive).

Bacterial Isolate Code	IAA Production μg mL^−^^1^	Root Colonization Ability log_10_ CFU/g	Phosphate Solubilization μg mL^−^^1^	Biofilm Production Activity OD_570_	ACC Deaminase (mmol α-Ketobutyrate mg^−1^ protein h^−1^)	Nitrogen Fixation
*Micrococcus luteus*	8.77 ± 0.34	6.54 ± 0.11	10.23 ± 0.31	1.38 ± 0.07	860.32 ± 0.25	+++
*Enterobacter cloacae*	8.69 ± 0.40	6.49 ± 0.31	7.03 ± 0.32		811.42 ± 0.54	++

**Table 3:** Morphological and biochemical characteristics of the most potent plant growth-promoting endophytic bacterial isolate.

Bacterial Isolate Code	Gram Reaction	Urease Activity	Catalase Test	Oxidase Test	Nitrate Reduction	Citrate Utilization	H_2_S Production	Indole	MR	VP
*Micrococcus luteus*	+	+	+	+	−	−	−	−	−	−
*Enterobacter cloacae*	−	+	+	−	+	+	−	−	−	+

+ (positive); − (negative).

**Table 4:** Heavy metal resistance behavior of the most potent plant growth promoting endophytic bacterial isolate; +++ (normal growth); ++ (moderate growth); + (weak growth); − (no growth).

Bacterial Isolate Code	Heavy Metal Tolerance												
	Control	Cd (μM)						Ni (μM)					
		3.125	6.25	12.5	25	50	100	3.125	6.25	12.5	25	50	100
*Micrococcus luteus*	+++	+++	+++	++	+	+	−	+++	+++	++	+	+	−
*Enterobacter cloacae*	+++	+++	+++	++	+	+	−	+++	+++	++	+	+	−

## Data Availability

Not applicable.
